# Acylglycerol kinase promotes cell proliferation and tumorigenicity in breast cancer via suppression of the FOXO1 transcription factor

**DOI:** 10.1186/1476-4598-13-106

**Published:** 2014-05-08

**Authors:** Xi Wang, Chuyong Lin, Xiaohui Zhao, Aibin Liu, Jinrong Zhu, Xinghua Li, Libing Song

**Affiliations:** 1 State Key Laboratory of Oncology in Southern China, Department of Experimental Research, Sun Yat-sen University Cancer Center, Guangzhou, Guangdong 510060, China; 2 Department of Breast Surgery, Cancer Center, Sun Yat-sen University, Guangzhou, Guangdong 510060, China; 3 Department of Biochemistry, Zhongshan School of Medicine, Sun Yat-sen University, Guangzhou, Guangdong 510080, China

**Keywords:** AGK, Breast cancer, Tumorigenicity, FOXO1

## Abstract

**Background:**

Acylglycerol kinase (AGK) is reported to be overexpressed in multiple cancers. The clinical significance and biological role of AGK in breast cancer, however, remain to be established.

**Methods:**

AGK expression in breast cancer cell lines, paired patient tissues were determined using immunoblotting and Real-time PCR. 203 human breast cancer tissue samples were analyzed by immunochemistry (IHC) to investigate the relationship between AGK expression and the clinicopathological features of breast cancer. Functional assays, such as colony formation, anchorage-independent growth and BrdU assay, and a xenograft tumor model were used to determine the oncogenic role of AGK in human breast cancer progression. The effect of AGK on FOXO1 transactivity was further investigated using the luciferase reporter assays, and by detection of the FOXO1 downstream genes.

**Results:**

Herein, we report that AGK was markedly overexpressed in breast cancer cells and clinical tissues. Immunohistochemical analysis showed that the expression of AGK significantly correlated with patients’ clinicopathologic characteristics, including clinical stage and tumor-nodule-metastasis (TNM) classification. Breast cancer patients with higher levels of AGK expression had shorter overall survival compared to patients with lower AGK levels. We gained valuable insights into the mechanism of AGK expression in breast cancer cells by demonstrating that overexpressing AGK significantly enhanced, whereas silencing endogenous AGK inhibited, the proliferation and tumorigenicity of breast cancer cells both *in vitro* and *in vivo*. Furthermore, overexpression of AGK enhanced G1-S phase transition in breast cancer cells, which was associated with activation of AKT, suppression of FOXO1 transactivity, downregulation of cyclin-dependent kinase inhibitors *p21*^*Cip1*^ and *p27*^*Kip1*^ and upregulation of the cell cycle regulator *cyclin D1*.

**Conclusions:**

Taken together, these findings provide new evidence that AGK plays an important role in promoting proliferation and tumorigenesis in human breast cancer and may serve as a novel prognostic biomarker and therapeutic target in this disease.

## Background

Breast cancer is the most common malignancy and leading cause of cancer-related deaths in females worldwide [1]. Several lines of evidence showed that multiple proteins are dysregulated in primary tumors and are associated with the development and progression of breast cancers [2-4]. Therefore, understanding the roles and molecular mechanisms of these proteins may provide new insights into the physiology and pathology of cancer and enable the development of novel and effective anticancer therapeutics.

FOXO1 is a member of the forkhead box-containing O subfamily (FOXO) family of transcription factors, which play vital roles in a variety of biological processes, including cell cycle arrest, cell death, apoptosis, stress response, cellular differentiation and metabolism [5,6]. For instance, ectopically expressing FOXO1 transcriptionally upregulates cell-cycle inhibitors, such as *p21*^*Cip1*^ and *p27*^*Kip1*^, and downregulate cell-cycle regulators, such as *cyclin D1* and *cyclin D2*, which result in G1/S arrest of cells [7-9]. Activation of FOXO1 could induce apoptosis through inducing expression of pro-apoptotic proteins, such as Puma, Bim, TRAIL and Fas ligand (FasL) [10-13]. FOXO1 has also been implicated in DNA repair mechanisms through upregulation of *GADD45a* by directly binding to the *GADD45* promoter [14]. Conversely, FOXO1 expression is found to be downregulated in multiple human cancers, including prostate cancer, endometrial carcinoma, glioblastoma and breast cancer [15-18]. Therefore, FOXO1 is considered to be as a putative tumor suppressor, and better understanding of the mechanisms that regulate FOXO1 activity may provide clues of novel targets for therapeutic intervention.

Acylglycerol kinase (AGK) is found to be abundantly expressed in the heart, muscle, kidney and brain [19]. By acting as a lipid kinase, it catalyzes the phosphorylation of acylglycerols to generate lysophosphatidic acid (LPA) [19-22], which is a potent lipid mediator that regulates a number of biological processes [23-25]. Recently, AGK is reported to be overexpressed in prostate cancer and esophageal squamous cell carcinoma (ESCC) [19,26,27]. Bektas et al. reported that AGK was upregulated in prostate, uterine, cervical and stomach cancers, and induced proliferation and migration in prostate cancer cells [19]. Chen et al. showed that overexpression of AGK promoted stem cell-like phenotypes in human ESCC both *in vivo* and *in vitro* and was correlated with progression and poor prognosis in ESCC [26]. In addition, Nouh et al. found that AGK expression was significantly correlated with primary Gleason grade of prostate cancer foci and prostate capsular invasion [27]. These findings have provided substantial evidence to show that AGK might contribute to the progression and development of cancer. However, the clinical significance and biological role of AGK in human breast cancer remain unclearly.

In this study, we found that AGK was markedly overexpressed in breast cancer cells and clinical tissue samples. Overexpressing AGK dramatically promoted the proliferation and tumorigenicity of breast cancer cell both *in vitro* and *in vivo*, whereas silencing AGK had the converse effect. Taken together, our findings suggest that AGK functions as an oncoprotein during breast cancer progression.

## Results

### AGK overexpression correlates with progression and poor prognosis in breast cancer

**Figure 1:**
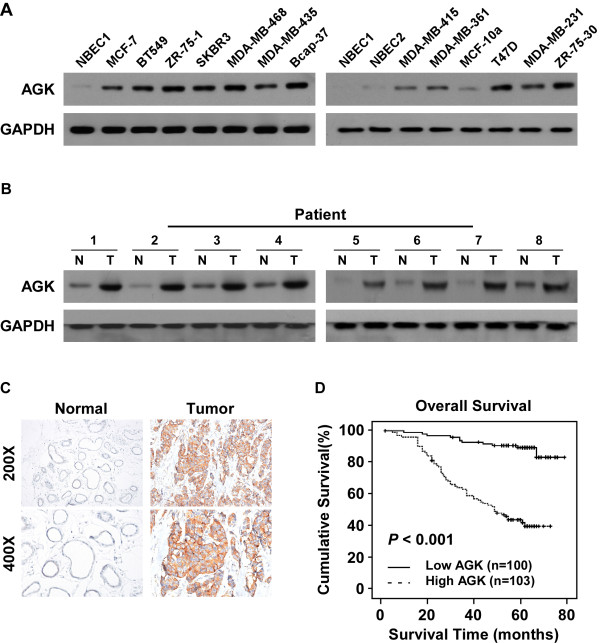
**AGK is upregulated in breast cancer. (A and B)** Western blotting analysis of AGK expression in normal breast epithelial cells (NBECs) and 12 breast cancer cell lines **(A)** and in eight matched primary breast cancer tissues (T) and adjacent noncancerous tissues (N) **(B)**. GAPDH was used as a loading control. **(C)** Immunohistochemical analysis of AGK protein expression in normal breast and primary tumor tissues. **(D)** Kaplan-Meier overall survival curves and univariate analyses (log-rank) comparing breast cancer patients with low (*n* = 103) and high (*n* = 100) AGK-expressing tumors (*P* < 0.001).

Western blotting and real-time PCR analyses were performed and showed that AGK mRNA and protein expression were markedly upregulated in all tested breast cancer cell lines compared to primary normal breast epithelial cells (NBECs) (Figure 1A and Additional file 1: Figure S1A). Consistently, we found that AGK expression was higher in eight human breast cancer tissues than the paired adjacent non-tumor tissues (Figure 1B and Additional file 1: Figure S1B). These results indicate that AGK expression is upregulated in breast cancer.

To investigate the relationship between AGK expression and the clinicopathological features of breast cancer, 203 human breast cancer tissue samples were analyzed by IHC. Consistently, IHC analysis indicated that AGK was markedly upregulated in breast cancer samples (Figure 1C). Furthermore, statistical analysis of the results revealed that AGK expression was strongly associated with the clinical stage (*P* < 0.001), T classification (*P* < 0.001), N classification (*P* < 0.001) and M classification (*P* = 0.003) (Additional file 2: Table S2). Kaplan-Meier survival curves and log-rank test showed that AGK expression was significantly correlated with overall survival (OS) in breast cancer (*P* < 0.001; Figure 1D). Similar results were obtained between patients in clinical stage I–II and III–IV subgroups (Additional file 3: Figure S2A-B). Furthermore, univariate and multivariate analyses indicated that clinical TNM classification and AGK expression were independent prognostic factors in breast cancer (Additional file 2: Table S3), suggesting that AGK might serve as a prognostic indicator of survival in patients with breast cancer.

### AGK promotes the proliferation of breast cancer cells

**Figure 2:**
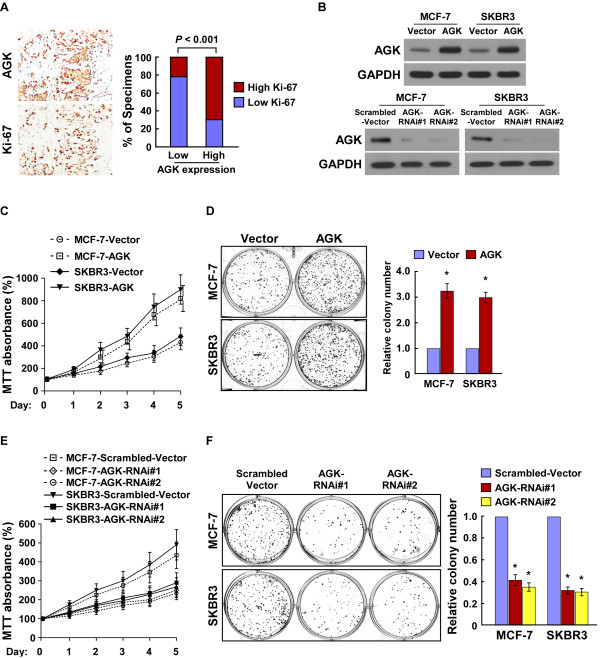
**AGK plays a key role in breast cancer cell proliferation and tumorigenicity. (A)** AGK expression levels significantly correlated with Ki-67 expression in human breast cancer tissues (n = 203; *P* < 0.001). Two representative cases are shown (left) and percentage of specimens with low or high AGK expression, relative to the levels of Ki-67 staining (right). **(B)** Western blotting analysis of AGK expression in AGK-infected MCF-7 and SKBR3 cells (upper panel) and AGK-silenced MCF-7 and SKBR3 cells (lower panel). GAPDH was used as a loading control. **(C-F)** MTT assay **(C, E)** and colony formation assay **(D, F)** indicated that the growth rate increased in AGK-overexpressing cells and decreased in AGK-silenced cells. The absorbance at day 1–5 was normalized to the absorbance at day 0 used as control (100%). The number of colonies was quantified in the colony formation assay. Each bar represents the mean ± SD of three independent experiments. *P < 0.05.

Since AGK expression was correlated with clinical stage in breast cancer (*P* < 0.001; Additional file 2: Tables S2 and S3), we then examined the relationship between AGK and Ki-67 expression. As shown in Figure 2A and Additional file 2: Tables S2, the tumor areas with the high levels of AGK staining also showed strong Ki-67 expression, whereas areas with low AGK staining intensities exhibited weak Ki-67 signals (*P* < 0.001), suggesting that AGK might promote proliferation of breast cancer cells.

To further investigate the effect of AGK on the proliferation of breast cancer cells by examining gain and loss of function models (Figure 2B). MTT and colony formation assays showed that the proliferation rate of AGK-overexpressing cells was significantly higher than the corresponding vector-control cells, whereas silencing AGK drastically reduced cell proliferation (Figure 2B-F). These results provide strong evidence that AGK plays a critical role in the proliferation of breast cancer cells.

### AGK promotes the tumorigenicity of breast cancer cells both in vitro and in vivo

**Figure 3:**
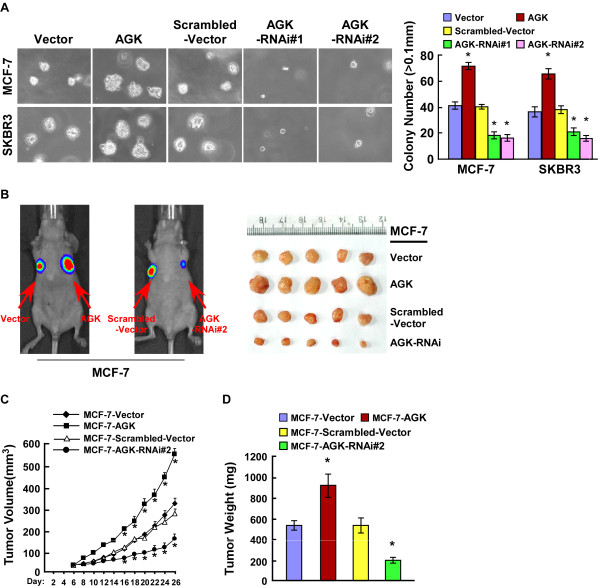
**AGK promotes the tumorigenicity of breast cancer cells *in vitro *and *in vivo*. (A)** Anchorage-independent growth assays of AGK-overexpressing cells and AGK-silenced cells. The number of colonies with a diameter larger than 0.1 mm was quantified after 10 days of culture. **(B-D)** Xenograft model in nude mice. MCF-7-AGK, MCF-7-AGK-RNAi and the respective control cells were inoculated into the fat-pad of nude mice (*n* = 5/group). **(B)**: Representative images of tumor-bearing mice (left panel) and images of the tumors from all mice in each group (right panel). **(C)** Tumor volumes were measured on the indicated days. **(D)** Mean tumor weights. Each bar represents the mean ± SD of three independent experiments. *P < 0.05.

Next, we investigated the effect of AGK on the tumorigenicity of breast cancer cells. As shown in Figure 3A, upregulation of AGK significantly increased, whereas downregulation of AGK decreased, the anchorage-independent growth ability of MCF-7 and SKBR3 cells in soft agar. Similarly, we observed that MCF-7/AGK tumors grew significantly faster than MCF-7/vector tumors, whereas the tumors formed by MCF-7/AGK-RNAi cells grew at a much slower rate than control MCF-7/Scrambled-vector tumors (Figure 3B-D). Collectively, these results indicate that AGK plays a significant role in the tumorigenicity of breast cancer cells both *in vitro* and *in vivo*.

### AGK regulates the G1-S phase transition in breast cancer cells

**Figure 4:**
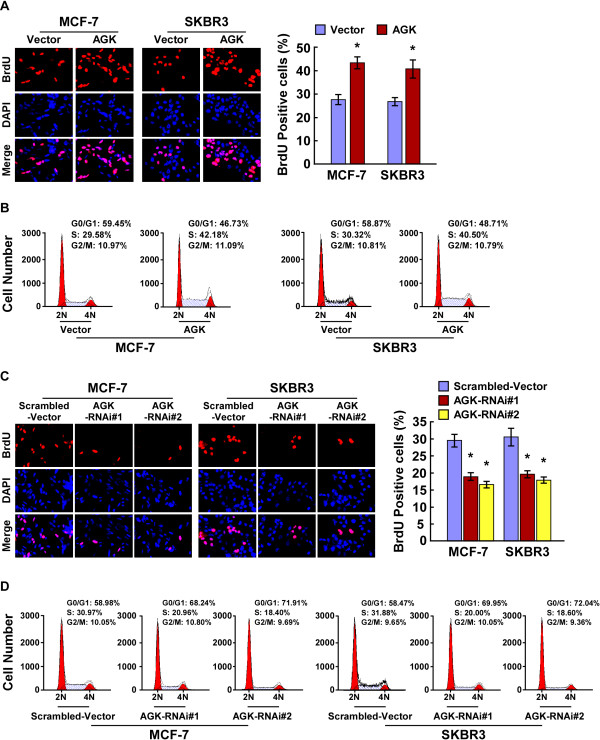
**AGK regulates the G1-S phase cell cycle transition in breast cancer cells. (A)** Representative micrographs (left) and quantification (right) of BrdU incorporation by the indicated cells. **(B)** Flow cytometric analysis of vector control and AGK-overexpressing cells. **(C)** Representative micrographs (left) and quantification (right) of BrdU incorporation by vector and AGK shRNA-infected cells. **(D)** Flow cytometric analysis of vector and AGK shRNA-infected cells. Each bar represents the mean ± SD of three independent experiments. *P < 0.05.

**Figure 5:**
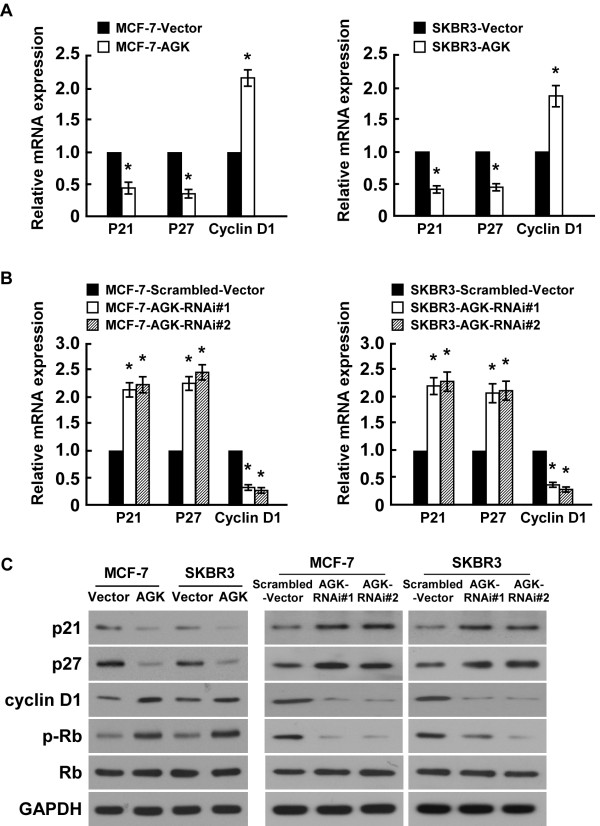
**AGK alters expression in G1-S phase cell-cycle regulators. (A and B)** Real-time PCR analysis of *p21*^*Cip1*^*, p27*^*Kip1*^ and *cyclin D1* mRNA expression in AGK-infected cells **(A)** or AGK-shRNA infected cells **(B)**. Gene expression levels were normalized to *GAPDH*. **(C)** Western blotting analysis of p21^Cip1^, p27^Kip1^, cyclin D1, p-Rb and total Rb protein expression in AGK-infected cells and AGK-shRNA infected cells; GAPDH was used as a loading control. Each bar represents the mean ± SD of three independent experiments. *P < 0.05.

To further explore the mechanism by which AGK promotes proliferation of breast cancer cells, BrdUrd incorporation and flow-cytometry assays were performed. As shown in Figure 4A-D, overexpressing AGK significantly increased, but silencing AGK reduced, the percentage of S phase cells. Further analyses by real-time RT-PCR and Western blotting showed that the expression of cyclin dependent kinases (CDK) inhibitors p21^Cip1^ and p27^Kip1^, at both mRNA and protein levels, were drastically reduced in AGK-overexpressing cells compared to control cells. This was accompanied by a concurrent increase in the levels of cell cycle regulators cyclin D1 and p-Rb (Figure 5A,C). Conversely, the expression of p21^Cip1^ and p27^Kip1^ significantly increased, whereas expression of cyclin D1 and p-Rb decreased, in AGK-silenced cells (Figure 5B-C). Collectively, our results suggest that AGK promotes cell cycle G1/S transition in breast cancer cells.

### AGK downregulates FOXO1 transactivity and activates AKT signaling pathway

**Figure 6:**
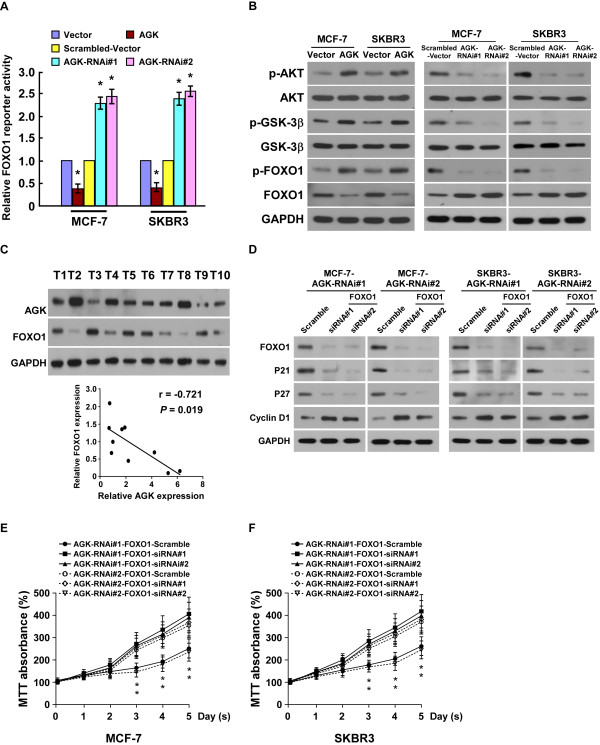
**AGK downregulates FOXO1 transaactivity and activates the AKT pathway. (A)** Relative FOXO1 reporter activity in AGK-infected cells, AGK-shRNA infected cells and control cells. **(B)** Western blotting analysis of phosphorylated-Akt (p-Akt), total Akt, phosphorylated-GSK-3β (p-GSK-3β), total GSK-3β, phosphorylated-FOXO1 (Ser256) and total FOXO1 proteins in MGF-7 and SKBR3 breast cancer cells. GAPDH was used as a loading control. **(C)** Expression (upper panel) and correlation analysis (lower panel) of AGK and FOXO1 expression in 10 freshly isolated human breast cancer samples. **(D)** Western blotting analysis of p21^Cip1^, p27^Kip1^, cyclin D1 and FOXO1 proteins in the indicated breast cancer cell lines. GAPDH was used as a loading control. **(E, F)** The MTT assay shows that silencing FOXO1 increased the proliferation of AGK-silenced cells as determined by an MTT assay. Error bars represent SD from three independent experiments. *P < 0.05.

Previous reports have demonstrated that FOXO1 transcriptionally regulates *p21*^*Cip1*^, *p27*^*Kip1*^ and *cyclin D1*[7,8], which prompted us to investigate whether AGK targeted these genes by modulating the transactivity of FOXO1. As shown in Figure 6A-B, the transactivity and expression level of FOXO1 significantly decreased in AGK-overexpressing cells and increased in AGK-silenced cells. We also found that AGK expression inversely correlated with FOXO1 in the ten freshly collected clinical breast cancer samples (r = -0.721, *P* = 0.019; Figure 6C). AKT kinase is known to play a key role in phosphorylating and repressing FOXO transcriptional activity [8]. As predicted, overexpressing AGK drastically increased, but silencing AGK decreased, the level of p-AKT and p-GSK-3β (Figure 6B), suggesting that AGK contributes to modulation of AKT/FOXO1 and AKT/GSK-3β signalings.

We further examined the role of FOXO1 in AGK-mediated cell proliferation. As shown in Additional file 4: Figure S3A, the luciferase activity of the FOXO1 reporter was significantly decreased in AGK-silenced cells after FOXO1 siRNA(s) transfection. Additional knockdown of FOXO1 in AGK-silenced cells decreased p27 ^Kip1^ and p21^Cip1^ expression but increased cyclin D1 expression (Figure 6D and Additional file 4: Figure S3B). Furthermore, MTT and colony formation assays showed that silencing FOXO1 restored the growth rate of AGK-silenced cells (Figure 6E-F and Additional file 4: Figure S3C). Taken together, our results suggest that FOXO1 plays a critical role in the pro-proliferative effect of AGK on breast cancer cells.

## Discussion

The key findings presented in this study suggest that AGK is markedly upregulated in breast cancer cells and its ectopic expression promotes the proliferation and tumorigenicity of breast cancer cells both *in vitro* and *in vivo*. The mechanistic basis for the pro-proliferative effect of AGK might be linked to activation of the AKT signaling pathway and subsequent inhibition of FOXO1 transcriptional activity, which would lead to altered expression of cell-cycle related genes, including the downregulation of CDK inhibitors, *p21*^*Cip1*^*p27*^*Kip1*^ and upregulation of *cyclin D1*. Our results have provided new insights into the role of AGK in the progression of human breast cancer, indicating that targeting AGK may offer a novel therapeutic strategy in the treatment of patients with breast cancer.

Although AGK has been shown to be overexpressed in several types of cancer and has been associated with cancer progression and development [19,26,27], its clinical significance and biological role in human breast cancer remains unclear. In this study, we found that AGK expression was upregulated in a large cohort of human breast cancer tissues, and was significantly correlated with the clinicopathologic characteristics of breast cancer, including clinical stage and TNM classification. Furthermore, survival analyses showed that patients with higher levels of AGK expression had shorter overall survival time compared to those with lower AGK expression, suggesting that AGK may represent a novel predictor for prognosis and survival in breast cancer. Several reports have also provided evidence that AGK is involved in the survival and motility of malignant phenotypes of cancer cells and the development of cancer stem cells [19,26]. In agreement with these reports, we found that AGK was strongly expressed in highly proliferative lesions of human breast cancer, as indicated by a significant correlation between AGK and Ki-67 expression (*P* < 0.001). Combined with our observation that upregulation of AGK was implicated in the proliferation and tumorigenicity of breast cancer cells both *in vivo* and *in vitro*. Therefore, our results support the proposal that AGK is a proliferation-promoting and oncogenic protein in breast cancer cells.

It has been established that FOXO1 functions as a tumor suppressor. Consistently, FOXO1 expression is reported to be downregulated in multiple cancer types, such as breast cancer, prostate cancer, glioblastoma and endometrial carcinoma [15-18]. FOXO1 has been demonstrated to be involved in multiple biological processes through transcriptional regulation of their downstream efforts, such as CDK inhibitors *p21*^*Cip1*^, *p27*^*Kip1*^and *p57*^*kip2*^, cell-cycle related genes *cyclin D1/D2*, and pro-apoptotic proteins, such as Puma, Bim, TRAIL and FasL [7-13,28,29]. We showed that depletion of FOXO1 restored the growth rate of *AGK*-silenced breast cancer cells, indicating that FOXO1 plays a critical role in the pro-proliferative effect of AGK on breast cancer cells.

By showing that AGK overexpression decreased the transactivation of FOXO1 and its downstream targets, we hypothesized that FOXO1 was involved in AGK-mediated proliferative mechanisms. It has been reported that phosphorylation of FOXO1 (Ser^256^) by AKT results in downregulation of FOXO1 transactivity *via* ubiquitin-proteasome-mediated degradation [30-32]. We observed similar effects, in that the levels of phospho-AKT and phospho-FOXO1 were increased in AGK-overexpressing cells and decreased in AGK-silenced cells. This suggested that the mechanism underlying AGK-mediated FOXO1 downregulation might be through activation of AKT. AKT is a major downstream effector of epidermal growth factor receptor EGFR and the non-receptor tyrosine kinase JAK2 [33,34]. Interestingly, it has been reported that AGK overexpression promotes aggressiveness in prostate cancer cells through activation of EGFR, and that upregulation of AGK promotes the stem cell-like phenotype in ESCC by sustaining JAK2 activity [15,22]. Meanwhile, we also observed that the phosphorylation level of GSK-3β, a downstream target protein of Akt, increased in the AGK-overexpressing cells and decreased in the AGK silenced cells. It has been reported that inactivation of GSK3β indicated by increased p-GSK3β was found in approximately half of the invasive mammary carcinomas, and significantly correlated with a worse clinical outcome [35]. Phosphorylation mediated suppression of GSK3β promotes breast tumor initiation and metastasis, and reduced phosphorylation of GSK3β efficiently inhibit cancer stem cell-like phenotypes in breast cancer [36,37]. Therefore, the role of AGK-modulation of GSK-3β activity in breast cancer cells is currently under investigation by our group.

## Conclusions

In summary, our results have demonstrated that AGK plays an important role in human breast cancer progression and have provided insights into the underlying mechanisms. Establishing the precise role played by AGK in breast cancer progression will not only advance our understanding of the biology of breast cancer but may offer a mechanism for a novel therapeutic strategy *via* suppression of AGK expression in breast cancer cells. Furthermore, our results suggest a potential role for AGK as a clinical predictor of disease progression, prognosis and survival in patients with breast cancer. Evaluating the molecular diagnostic ability of AGK in breast cancer is merited.

## Methods

### Cell lines

Primary normal breast epithelial cells (NBECs) were established as previously described [38]. Breast cancer cell lines, including MCF-7, BT-549, ZR-75-1, SKBR3, MDA-MB-468, MDA-MB-435, Bcap37, MDA-MB-415, MDA-MB-361, T47D, MDA-MB-231 and ZR-75-30 were cultured in DMEM medium (Gibco, Grand Island, NY) supplemented with 10% FBS (HyClone, Logan, UT).

### Patient information and tissue specimens

This study was conducted on a total of 203 paraffin-embedded, archived breast cancer samples, which had been histopathologically and clinically diagnosed at the Sun Yat-sen University Cancer Center from 1998 to 2006. Clinical and clinicopathological classification and stage were determined according to American Joint Committee on Cancer (AJCC) criteria [39] and summarized in Additional file 2: Table S1. Ethics approval and prior patient consent had been obtained from the Institutional Research Ethics Committee for the use of the clinical specimens for research purposes.

### Vectors and retroviral infection

The human AGK gene was PCR-amplified from cDNA and cloned into the pSin-EF2 lentiviral vector, and shRNAs targeting AGK were cloned into the pSuper-retro viral vector, as previously described [26]. Retroviral production and infection were performed as previously described [40]. Stable cell lines expressing AGK or AGK shRNAs were selected for 10 days with 0.5 μg/ml puromycin. The reporter plasmid for detecting the transcriptional activity of FOXO1 was generated as described previously [41].

### Immunohistochemistry (IHC)

Immunohistochemistry (IHC) and quantification of AGK expression were performed by two independent pathologists, as previously described [26]. Both sets of results were combined to give a mean score for further comparative evaluations. Briefly, the IHC score, or staining index (SI), was determined by combining the score for the percentage of positively-stained tumor cells with the grade of the staining intensity. The percentages of positively-stained tumor cells were scored as follows: 0, no positive tumor cells; 1, <10%; 2, 10%–35%; 3, 35%–75%; 4, >75%. The staining intensities were graded as follows: 1, no staining; 2, weak staining (light yellow); 3, moderate staining (yellow–brown); 4, strong staining (brown). We used this method to evaluate AGK expression in benign breast epithelia and malignant lesions. The possible scores were 0, 2, 3, 4, 6, 8, 9, 12 and 16; SI ≥8 was defined as high expression and SI <8 was defined as low expression.

### Western blotting

Western blotting was carried out according to standard methods as described previously [38], by using anti-AGK antibody (Epitomics, Burlingame, CA), anti-p21^Cip1^, anti-p27^Kip1^, anti-cyclin D1, anti-Rb, anti-phosphorylated-Rb, anti-AKT, anti-phosphorylated-AKT, anti-FOXO1, anti-phosphorylated-FOXO1 (Ser256) (Cell Signaling, Danvers, MA). The membranes were stripped and re-probed with an anti-GAPDH antibody (Sigma, Saint Louis, MI) as a loading control.

### MTT cell viability assay

Cells were seeded in 96-well plates at a density of 2 × 10^3^ cells/well. At each time point, cells were stained with 100 μl sterile MTT dye (0.5 mg/ml, Sigma) for 4 hours at 37°C, followed by removal of the culture medium and addition of 100 μl of dimethyl sulphoxide (Sigma). The absorbance was measured at 570 nm, with 655 nm as the reference wavelength. The absorbance at day 1–5 was normalized to the absorbance at day 0 used as control (100%). Each experiment was performed in triplicates.

### Colony formation assay

Cells were plated in 6-well plated (5 × 10^2^ cells) and cultured for 10 days. The colonies were stained with 1% crystal violet for 30 seconds after fixation with 4% formaldehyde for 5 minutes. Colonies were counted and the resultes were shown as the fold change compared to vector control cells.

### Anchorage-independent growth ability assay

Five hundred cells were trypsinized and suspended in 2 ml complete medium plus 0.3% agar (Sigma, Saint Louis, MI). The agar-cell mixture was plated on top of a bottom layer with 1% agar completed medium mixture. About 10 days, viable colonies that were larger than 0.1 mm were counted. The experiment was carried out for each cell line in triplicates.

### Bromodeoxyuridine (BrdU) labeling and immunofluorescence

Cells (5 × 10^4^) were plated on coverslips. After 24 hours, cells were incubated with BrdU for 1 h and stained with anti-BrdU antibody (Upstate, Billerica, MA) according to the manufacturer’s instruction. After washing three times with PBS containing 1% Triton X-100, the cells were treated with anti-mouse TRITC fluorescent conjugated secondary antibodies to visualize anti-BrdU labeled cells. BrdU positive cells were counted under a laser scanning microscope (Axioskop 2 plus; Carl Zeiss Co. Ltd.) in ten random chosen fields from three independent samples. Percentage of BrdU positive cells was then calculated, and the results are presented as the mean ± SD.

### Flow cytometry

Cells were harvested, washed with cold PBS, and processed for cell cycle analysis by using flow cytometry. Briefly, the cells were fixed in 75% ethanol and stored at -20°C overnight for later analysis. The fixed cells were centrifuged at 1,000 rpm for 5 min and washed with cold PBS twice. RNase A (20 μg/ml final concentration) and propidium iodide staining solution (50 μg/ml final concentration) were added to the cells and incubated for 30 minutes at 37°C in the dark. Twenty thousand cells were analyzed by using a CytomicsTM FC 500 instrument (Beckman Coulter, USA) equipped with CXP software. Modfit LT 3.1 trial cell cycle analysis software was used to determine the percentage of cells in the different phases of the cell cycle.

### Xenografted tumor model

Female BALB/c nude mice (5 ~ 6 weeks of age, 18 ~ 20 g) were purchased from the Slac-Jingda Laboratory Animal (Hunan, China), and were housed in barrier facilities on a 12-hour light/dark cycle. All experimental procedures were approved by the Institutional Animal Care and Use Committee of Sun Yat-sen University. The BALB/c nude mice were randomly divided into 4 groups (n = 5/group). A 0.72 mg E2 60-day release pellet (Innovative Research of America) was implanted subcutaneously on the dorsal side of each mouse 1 day before tumor cell implantation to support the growth of the estrogen-dependent MCF-7 cell derived tumors. For tumor cell implantation, MCF-7-AGK or MCF-7-AGK-RNAi or their respective control cells (2 × 10^6^) in 200 μl of the mixture were injected into the mammary fat pads of mice. Tumors were examined twice weekly; length, width, and thickness measurements were obtained with calipers and tumor volumes were calculated. On day 26, animals were euthanized, and tumors were excised and weighed.

### Statistical analysis

Statistical analyses were performed using the SPSS version 13.0 statistical software package. Statistical tests for data analysis included log rank test, *χ*2 test, spearman-rank correlation test and Student’s 2-tailed t test. Multivariate statistical analysis was performed using a Cox regression model. Data represent mean ± SD. A *P*-value < 0.05 was considered statistically significant.

## Abbreviations

AGK: Acylglycerol kinase; IHC: Immunochemistry; TNM: Tumor-nodule-metastasis; FOXO: Forkhead box-containing O subfamily; FASL: Fas ligand; LPA: Lysophosphatidic acid; OS: Overall survival; CDK: Cyclin dependent kinases; NBECs: Normal breast epithelial cells; BrdU: Bromodeoxyuridine.

## Competing interests

The authors declare that they have no competing interests.

## Authors’ contributions

XW, CL and XZ were responsible for most experiments, data collection and analysis. AL and JZ were responsible for conducting the data analysis and Luciferase assay. XL was responsible for Real-time PCR assay. LS was responsible for experimental design, supervised the project and wrote the manuscript. All authors have read and approved the final manuscript.

## Supplementary Material

Additional file 1: Figure S1 AGK is upregulated in breast cancer. **(A-B)** Real-time PCR analysis of *AGK* mRNA expression in NBECs and twelve breast cancer cell lines **(A)**, and in matched primary breast cancer tissues (T) and the adjacent noncancerous tissues (N) **(B)**. Expression levels were normalized to *GAPDH*. Error bars represent SD from three independent experiments. *P < 0.05. Click here for file

Additional file 2: Table S1 Clinicopathological characteristics of patient samples and expression of AGK in Breast Cancer. **Table S2.** Correlation between AGK expression and clinicopathologic characteristics of Breast Cancer. **Table S3.** Univariate and multivariate analyses of various prognostic parameters in patients with Breast cancer Cox-regression analysis. Click here for file

Additional file 3: Figure S2 **(A-B)** Kaplan-Meier overall survival curves and log-rank test in breast cancer patients stratified by clinical stage I-II (*n* = 131) **(A)** and clinical stage III-IV (*n* = 72) **(B)**. The curves compare patients with high and low expression levels of AGK. Click here for file

Additional file 4: Figure S3 FOXO1 plays an important role in AGK-mediated proliferation. **(A)** Relative FOXO1 reporter activity in AGK-silenced MCF-7 and MDA-MB-231 cells after transfection with FOXO1 siRNA(s). **(B)** Real-time PCR analysis of p21^Cip1^, p27^Kip1^ and cyclin D1 mRNA expression in AGK-silenced MCF-7 and MDA-MB-231 cells after transfection with FOXO1 siRNA(s). Expression levels were normalized to *GAPDH*. (C) Quantification of colony formation assays in indicated cells. Error bars represent SD from three independent experiments. *P < 0.05. Click here for file
