# Supplementary figures and images for: Acylglycerol kinase promotes cell proliferation and tumorigenicity in breast cancer via suppression of the FOXO1 transcription factor
Source: Mol Cancer. 2014 May 8;13:106. doi: 10.1186/1476-4598-13-106 (PMC4028287; doi:10.1186/1476-4598-13-106)

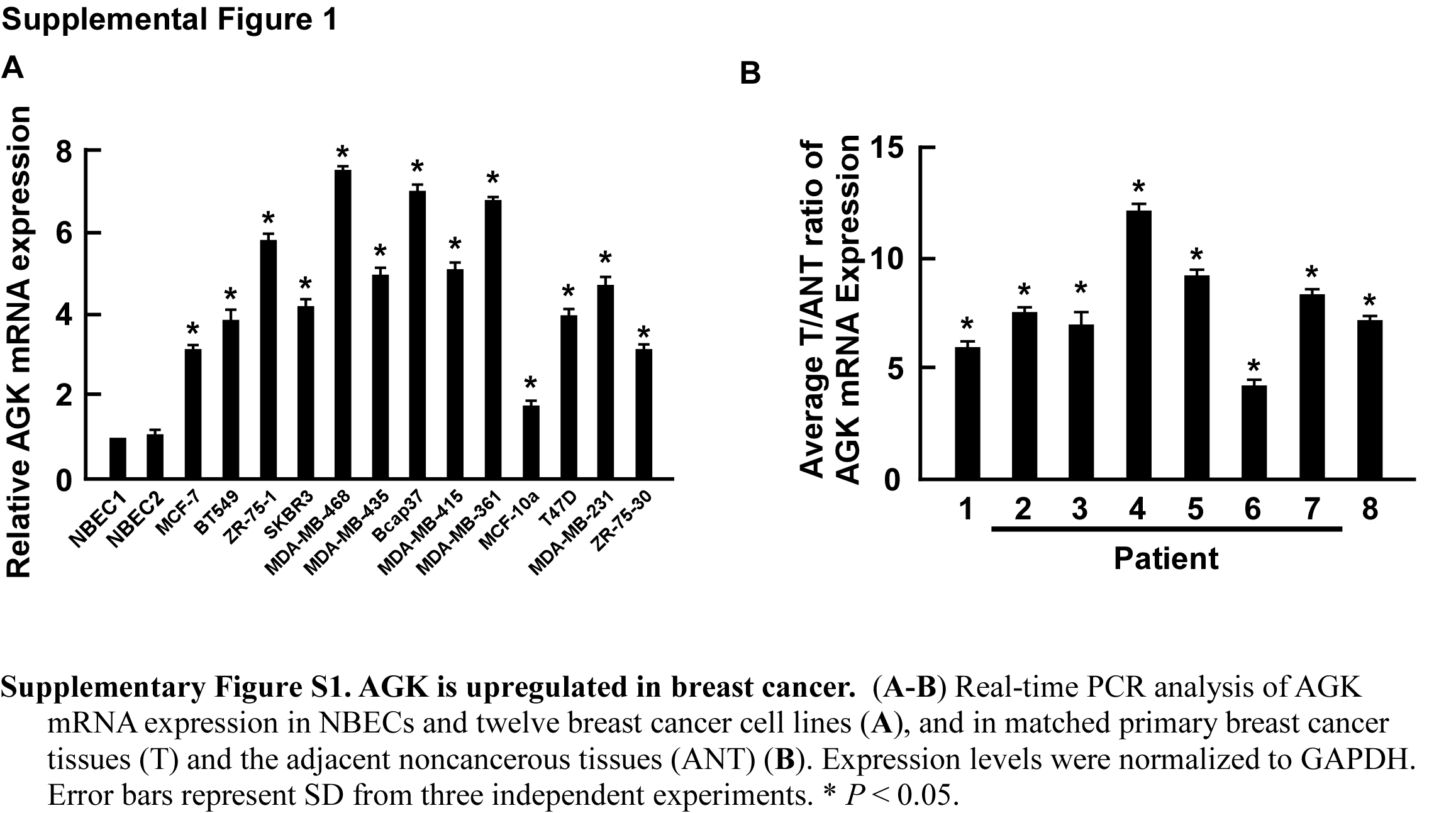

Supplement: Additional file 1: Figure S1 — AGK is upregulated in breast cancer. (A-B) Real-time PCR analysis of AGK mRNA expression in NBECs and twelve breast cancer cell lines (A), and in matched primary breast cancer tissues (T) and the adjacent noncancerous tissues (N) (B). Expression levels were normalized to GAPDH. Error bars represent SD from three independent experiments. *P < 0.05. [file 1476-4598-13-106-S1.tiff]

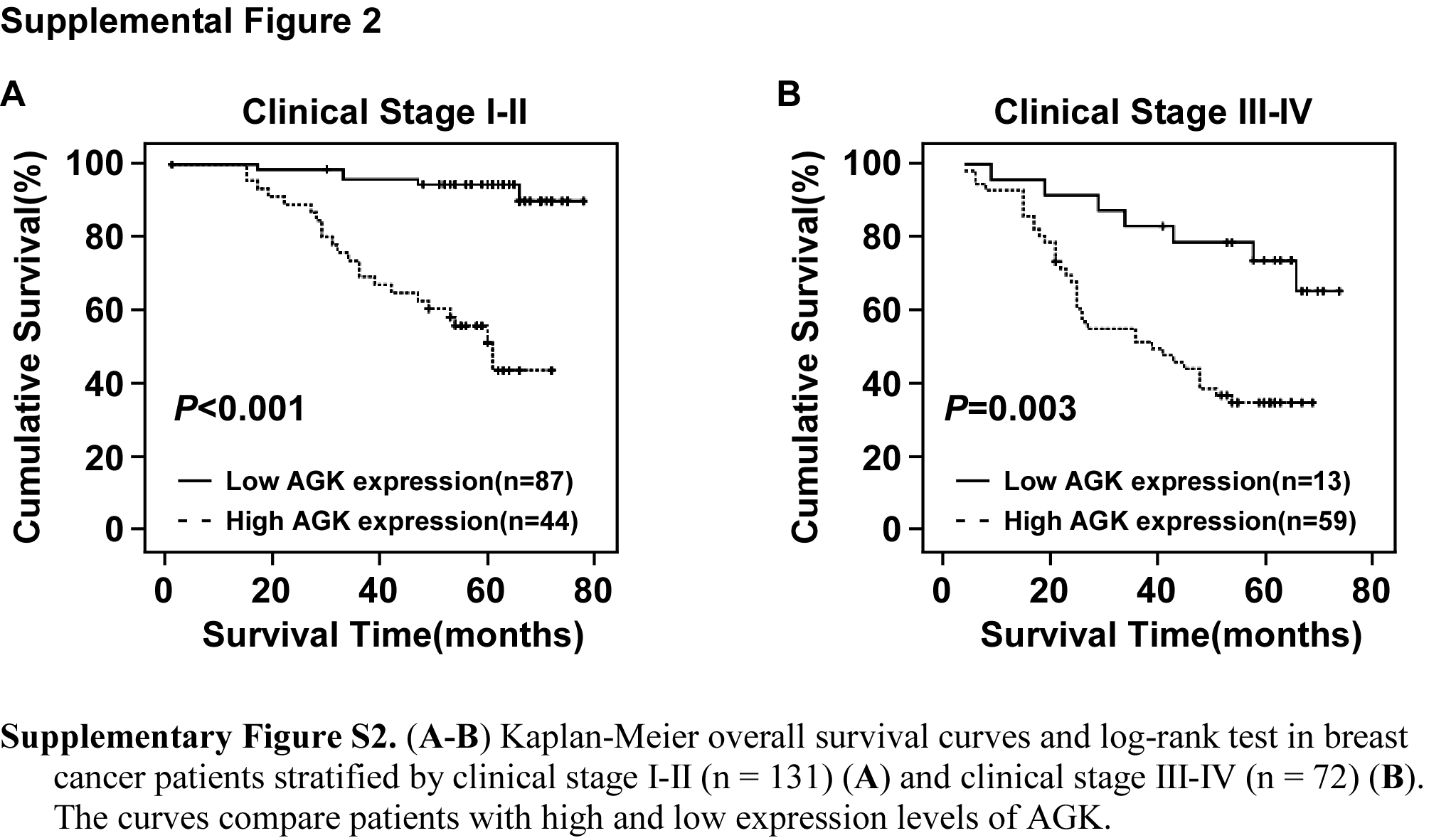

Supplement: Additional file 3: Figure S2 — (A-B) Kaplan-Meier overall survival curves and log-rank test in breast cancer patients stratified by clinical stage I-II (n = 131) (A) and clinical stage III-IV (n = 72) (B). The curves compare patients with high and low expression levels of AGK. [file 1476-4598-13-106-S3.tiff]

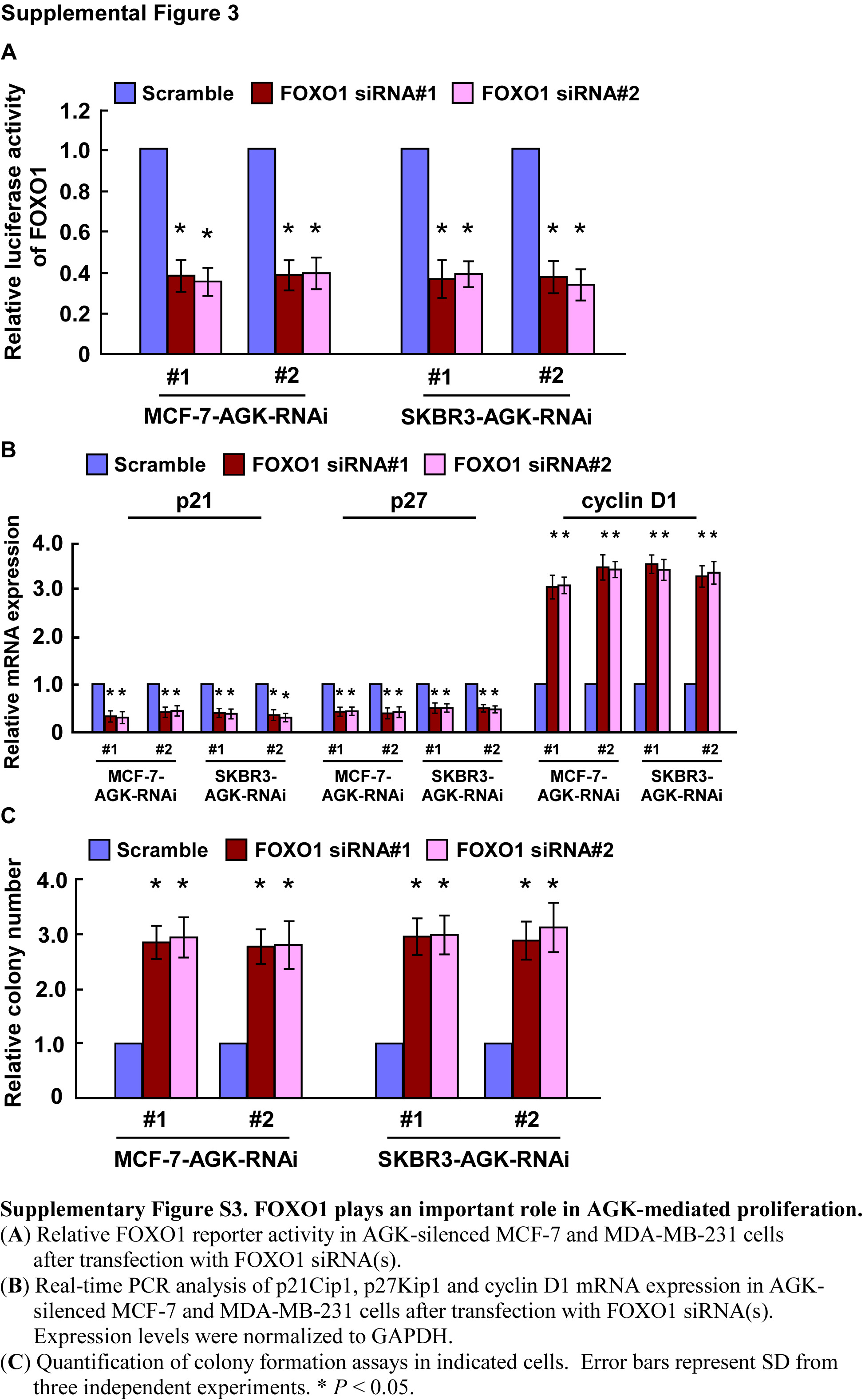

Supplement: Additional file 4: Figure S3 — FOXO1 plays an important role in AGK-mediated proliferation. (A) Relative FOXO1 reporter activity in AGK-silenced MCF-7 and MDA-MB-231 cells after transfection with FOXO1 siRNA(s). (B) Real-time PCR analysis of p21Cip1, p27Kip1 and cyclin D1 mRNA expression in AGK-silenced MCF-7 and MDA-MB-231 cells after transfection with FOXO1 siRNA(s). Expression levels were normalized to GAPDH. (C) Quantification of colony formation assays in indicated cells. Error bars represent SD from three independent experiments. *P < 0.05. [file 1476-4598-13-106-S4.tiff]
