# Supplementary material for: Acylglycerol kinase promotes cell proliferation and tumorigenicity in breast cancer via suppression of the FOXO1 transcription factor
Source: Mol Cancer. 2014 May 8;13:106. doi: 10.1186/1476-4598-13-106 (PMC4028287; doi:10.1186/1476-4598-13-106)
Supplement: Additional file 2: Table S1 — Clinicopathological characteristics of patient samples and expression of AGK in Breast Cancer. Table S2. Correlation between AGK expression and clinicopathologic characteristics of Breast Cancer. Table S3. Univariate and multivariate analyses of various prognostic parameters in patients with Breast cancer Cox-regression analysis. [file 1476-4598-13-106-S2.doc]

**Additional file 2: Table S1.** Clinicopathological characteristics of patient samples and expression of AGK in Breast Cancer

| **Gender** | Number of cases (%) |
| --- | --- |
| Male | 0(0.0%) |
| Female | 203(100.0%) |
| **Age (years)** |  |
| ≥47 | 94(46.3%) |
| <47 | 109(53.7%) |
| **Clinical Stage** |  |
| I | 27(13.3%) |
| II | 104(51.2%) |
| III | 45 (22.2%) |
| IV | 27(13.3%) |
| **T classification** |  |
| T0 | 4 (2.0%) |
| T1 | 46 (22.7%) |
| T2 | 93 (45.8%) |
| T3 | 37 (18.2%) |
| T4 | 23 (11.3%) |
| **N classification** |  |
| N0 | 75(37.0%) |
| N1 | 89(43.8%) |
| N2 | 37(18.2%) |
| N3 | 2(1.0%) |
| **M classification** |  |
| No | 176(86.7%) |
| Yes | 27(13.3%) |
| **Vital status (at follow-up)** |  |
| Alive | 133(65.5%) |
| Death (All Breast cancer-related) | 70(34.5%) |
| **Expression of AGK** |  |
| Low expression | 100(49.3%) |
| High expression | 103(50.7%) |
| **Expression of Ki-67** |  |
| Low expression | 108(53.2%) |
| High expression | 95(46.8%) |
| **Expression of Estrogen receptor** |  |
| 0 | 91(44.8%) |
| 1 | 93(45.8%) |
| 2 | 13(6.4%) |
| 3 | 6 (3.0%) |
| **Expression of Progestogen receptor** |  |
| 0 | 85(41.9%) |
| 1 | 90(44.3%) |
| 2 | 23(11.3%) |
| 3 | 4(2.0%) |
| 4 | 1(0.5%) |
| **Expression of HER2** |  |
| 0 | 27(26.0%) |
| 1 | 34(32.7%) |
| 2 | 22(21.1%) |
| 3 | 16(15.4%) |
| 4 | 5(4.8%) |

**Additional file 2: Table S2.** Correlation between AGK expression and clinicopathologic characteristics of Breast Cancer

| Characteristics | | AGK | | Chi-square test (*P* value) |
| --- | --- | --- | --- | --- |
|  |  | Low (%) | High (%) |  |
| **Age (years)** | ≥ 47 | 48(23.6) | 46 (22.7) | 0.633 |
|  | < 47 | 52(25.6) | 57(28.1) |  |
| **Clinical Stage** | I | 22(10.8) | 5(2.5) | < 0.001 |
|  | II | 65 (32.0) | 39 (19.2) |  |
|  | III | 7 (3.5) | 38 (18.7) |  |
|  | IV | 6 (3.0) | 21 (10.3) |  |
| **T classification** | T0 | 3(1.5) | 1(0.5) | < 0.001 |
|  | T1 | 34(16.7) | 12 (5.9) |  |
|  | T2 | 48(23.6) | 45 (22.2) |  |
|  | T3 | 10 (4.9) | 27 (13.3) |  |
|  | T4 | 5 (2.5) | 18 (8.9) |  |
| **N classification** | N0 | 50 (24.6) | 25 (12.3) | < 0.001 |
|  | N1 | 46(22.7) | 43 (21.2) |  |
|  | N2 | 4(2.0) | 33 (16.2) |  |
|  | N3 | 0(0.0) | 2(1.0) |  |
| **M classification** | No | 94(46.3) | 82(40.4) | 0.003 |
|  | Yes | 6(3.0) | 21(10.3) |  |
| **Ki-67** | Low | 78(38.4) | 30(14.8) | < 0.001 |
|  | High | 22(10.8) | 73(36.0) |  |
| **ER** | 0 | 47(23.2) | 44(21.6) | 0.641 |
|  | 1 | 44(21.6) | 49(24.1) |  |
|  | 2 | 5(2.5) | 8(4.0) |  |
|  | 3 | 4(2.0) | 2(1.0) |  |
| **PR** | 0 | 39 (19.2) | 46(22.7) | 0.750 |
|  | 1 | 45(22.15) | 45(22.15) |  |
|  | 2 | 13(6.4) | 10(4.9) |  |
|  | 3 | 2(1.0) | 2(1.0) |  |
|  | 4 | 1(0.5) | 0(0) |  |
| **HER2** | 0 | 17(16.3) | 10(9.6) | 0.070 |
|  | 1 | 21(20.2) | 13(12.5) |  |
|  | 2 | 6(5.8) | 16(15.4) |  |
|  | 3 | 7(6.7) | 9(8.7) |  |
|  | 4 | 2(1.9) | 3(2.9) |  |

**Additional file 2: Table S3.** Univariate and multivariate analyses of various prognostic parameters in patients with Breast cancer Cox-regression analysis

|  | Univariate analysis | | | Multivariate analysis | | |
| --- | --- | --- | --- | --- | --- | --- |
|  | No.  patients | *P* | Regression coefficient (SE) | *P* | Relative  risk | 95% confidence  interval |
| **Clinical stage** |  |  |  |  |  |  |
| I | 27 | < 0.001 | 0.907(0.126) | < 0.001 | 3.902 | 2.082-7.315 |
| II | 104 |  |  |  |  |  |
| III | 45 |  |  |  |  |  |
| IV | 27 |  |  |  |  |  |
| **T classification** |  |  |  |  |  |  |
| T0 | 4 | < 0.001 | 1.010(0.130) | 0.013 | 1.444 | 1.079-1.931 |
| T1 | 46 |  |  |  |  |  |
| T2 | 93 |  |  |  |  |  |
| T3 | 37 |  |  |  |  |  |
| T4 | 23 |  |  |  |  |  |
| **M classification** |  |  |  |  |  |  |
| No | 176 | 0.002 | 0.905(0.293) | < 0.001 | 0.139 | 0.051-0.382 |
| Yes | 27 |  |  |  |  |  |
| **Expression of AGK** |  |  |  |  |  |  |
| Low expression | 100 | < 0.001 | 1.948(0.322) | 0.005 | 2.652 | 1.333-5.278 |
| High expression | 103 |  |  |  |  |  |
